# Promising Molecular Targets for Pharmacological Therapy of Neurodegenerative Pathologies

**DOI:** 10.32607/actanaturae.10925

**Published:** 2020

**Authors:** M. E. Neganova, Yu. R. Aleksandrova, V. O. Nebogatikov, S. G. Klochkov, A. A. Ustyugov

**Affiliations:** Institute of Physiologically Active Compounds of the Russian Academy of Sciences, Moscow region, Chernogolovka, 142432 Russia

**Keywords:** neuroinflammation, oxidative stress, histone deacetylases, proteinopathy, aggregation of pathogenic proteins

## Abstract

Drug development for the treatment of neurodegenerative diseases has to confront numerous problems occurring, in particular, because of attempts to address only one of the causes of the pathogenesis of neurological disorders. Recent advances in multitarget therapy research are gaining momentum by utilizing pharmacophores that simultaneously affect different pathological pathways in the neurodegeneration process. The application of such a therapeutic strategy not only involves the treatment of symptoms, but also mainly addresses prevention of the fundamental pathological processes of neurodegenerative diseases and the reduction of cognitive abilities. Neuroinflammation and oxidative stress, mitochondrial dysfunction, dysregulation of the expression of histone deacetylases, and aggregation of pathogenic forms of proteins are among the most common and significant pathological features of neurodegenerative diseases. In this review, we focus on the molecular mechanisms and highlight the main aspects, including reactive oxygen species, the cell endogenous antioxidant system, neuroinflammation triggers, metalloproteinases, α-synuclein, tau proteins, neuromelanin, histone deacetylases, presenilins, etc. The processes and molecular targets discussed in this review could serve as a starting point for screening leader compounds that could help prevent or slow down the development of neurodegenerative diseases.

## INTRODUCTION

The development of effective therapeutic approaches to the treatment of neurological disorders is one of the most daunting challenges of modern biomedicine. The central issue is the absence of drugs that affect the disease pathogenesis. At the same time, the number of patients with the most common neurodegenerative diseases (NDD), such as Alzheimer’s disease and other forms of dementia, is estimated at approximately 30–35 million and doubles every 10 years worldwide[1]. The figure is expected to reach 70 million people in the next 10 years [2]. Total worldwide treatment expenses for patients with neurological disorders in 2015 amounted to US$ 818 billion and could potentially jump to US$ 2 trillion by 2030 [2]. About one hundred drugs for the treatment of Alzheimer’s disease, including vaccines, undergo clinical trials every year [3]. However, despite the vast resources involved, no new drug has been brought to market since 2003. An analysis of current developments in the field of new medicinal products for NDD suggests that most of the activity is focused on a search for multi-target compounds that affect the key aspects of pathogenesis [4]. Proteinopathy processes (pathological aggregation of specific proteins in the brain), mitochondrial dysfunction, neuroinflammatory processes, and dysfunction of histone deacetylases (HDACs), which serve as regulatory elements in the expression of the genes related to neurological disorders, are among the key pathological features that need addressing.

## PROBLEMS AND TARGETS IN THE TREATMENT OF NEURODEGENEATIVE DISEASES

Today, about a billion people worldwide suffer from neurodegenerative diseases. The most common are Alzheimer’s disease, Parkinson’s disease, and amyotrophic lateral sclerosis. They can occur as a result of a combination of genomic, epigenomic, metabolic and environmental factors. The risk of developing most neurodegenerative diseases increases with age, resulting in a progressive neurodegenerative process (in some cases due to the death of neuronal cells in various brain regions, in other cases as a result of motoneuron death), as well as neuroinflammatory processes. Currently, the available treatment methods cannot prevent or arrest the progression of neurodegenerative diseases. No basic therapy which could accrue significant benefits to patients with detrimental disorders has been developed so far. Modern treatment methods can only improve a patient’s condition affecting symptom manifestation with cognitive impairments and motor body functions temporarily. However, with the improvements in our quality of life, average life expectancy has increased considerably, and so has the number of age-related diseases. Hence, the detection of new targets for drug action, the development of new synthesis methods, and target-oriented selection of potential neuroprotectors are a priority both in modern medical chemistry and healthcare in general.

**Fig. 1:**
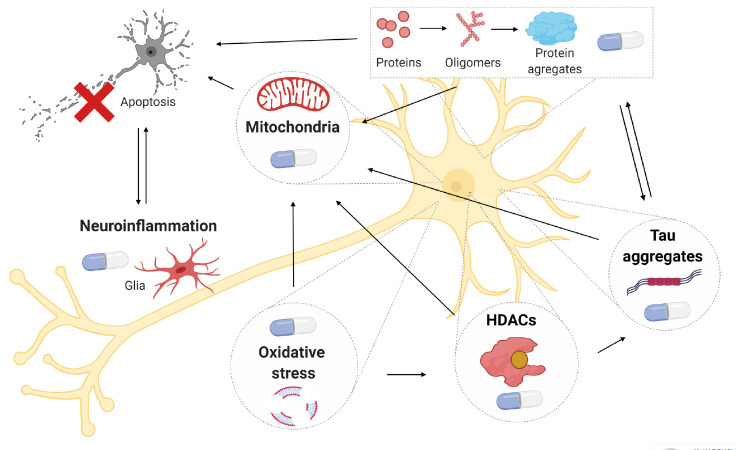
Molecular targets for pharmacological effects in the treatment of neurodegenerative diseases

In neurodegenerative diseases, the progression of pathology begins many years before the appearance of the first evident symptoms of the disease. Numerous studies have suggested that there are a number of common events among pathological conditions which can explain why an ageing brain is vulnerable to neurodegeneration. Physiological neuronal processes such as endosomal-lysosomal autophagy, neuroinflammatory reactions, mitochondrial homeostasis, and proteostasis are beyond systemic control in neurodegenerative diseases. The changes that occur in the redox cell balance and mitochondrial functioning, the impairment of the expression and activity of epigenetic enzymes and the increased pool of aggregated proteins with an impaired tertiary structure (Aβ, α-synuclein, etc.) are the main indicators of the development of neurodegenerative diseases (*Fig. 1*).

Oxidative stress and, in particular, peroxidation of membrane lipids, impairment of endogenous antioxidant mechanisms (glutathione system), and mitochondrial dysfunction (suppression of the activity of complex I and complex IV of the respiratory chain – cytochrome-c-oxidase) are inter-related and reinforce each other, leading to neurodegenerative processes [5, 6, 7]. Moreover, dead cell remnants and the aggregated proteins released into the extracellular environment from the neuron provoke glial activation and the release of cytokines and free radicals, leading to neuronal death, which triggers an additional pathological process: neuroinflammation. Pharmacological treatment of the abovementioned manifestations of early neurodegeneration stages could arrest the disease’s progression. This is therefore highly important for the medical treatment of neurodegeneration (*Fig. 1*).

**Role of oxidative stress in the development of a neurodegenerative process**

**Fig. 2:**
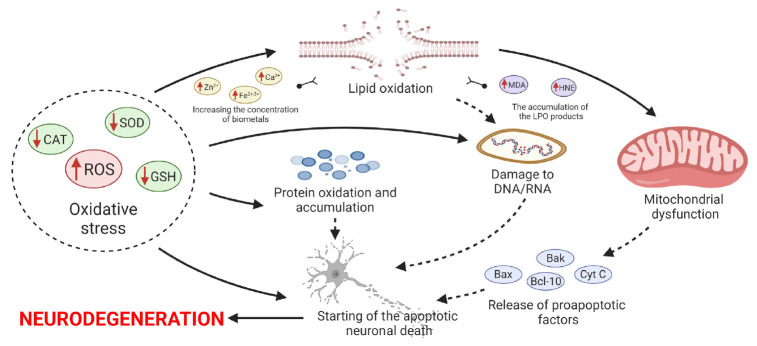
Oxidative stress in the development of neurodegenerative diseases. The increase of oxidative processes is associated with hyperproduction of reactive oxygen species and a decreased activity of the endogenous antioxidant defence system of cells leading to oxidative damage to lipids, proteins, and DNA/RNA, which triggers a cascade of apoptotic neuronal cell death and promotes neurodegeneration

Oxidative stress, a process which occurs as a result of the impairment of the pro-oxidant-antioxidant balance that promotes oxidative species, leading to potential damage to the cell [8, 9] and is the result of excessive accumulation of reactive oxygen species (ROS), as well as a decreased activity of the antioxidant system of cell defence, has always played a pivotal role in neurodegenerative diseases (Alzheimer’s disease, Parkinson’s disease, and others), including ageing [10-13]. The concentration of reactive oxygen species in physiological conditions is maintained at a relatively low level thanks to the activity of endogenous antioxidant mechanisms such as the glutathione system, superoxide dismutase, catalase, etc. [14]. However, with age and due to genetic and ecological risk factors, the redox system becomes unbalanced, resulting in the production of reactive oxygen species [15, 16]. Though ROS in moderate concentrations plays an important role in physiological processes (for example, in the regulation of signalling pathways and induction of the mitogenic response), its overproduction and imbalance in the endogenous antioxidant defence system leads to oxidative damage such as post-translation modifications and the oxidation of proteins, lipids and DNA/RNA, which are the shared features of many NDDs [17, 18]. Thus, patients with various neurological disorders (in particular, Alzheimer’s disease and Parkinson’s disease) suffer from ROS overproduction in the brain [19, 20], leading to increased peroxidation of membrane lipids through the action of free radicals, an elevated content of malone-dialdehyde in the system, excessive accumulation of metals with variable valency, and mitochondrial dysfunction with a subsequent release of apoptogenic factors and further neuronal apoptosis (*Fig. 2*) [21, 22].

It should be noted that such neuronal susceptibility to oxidative damage has several reasons [23, 16]. Membrane lipids in the brain contain a large amount of polyunsaturated fatty acids, which are prone to free radical attack and lipid peroxidation. In addition, active neurons also exhibit a high level of oxygen consumption, exacerbating therefore ROS production [24]. Moreover, it has also been shown that the brain contains quite a small amounts of enzymes for its own antioxidant cell protection, which play an important role in the metabolism of free radicals [25].

Malondialdehyde, 4-hydroxy-trans-2,3-nonenal, acrolein, and F2-isoprostanes are known oxidative stress markers that are routinely encountered in the brain and cerebrospinal fluid of patients with Alzheimer’s. Greilberger *et al. *investigated the blood of healthy individuals and that of patients with neurodegenerative disorders (mild cognitive disorders and Alzheimer’s), and they discovered that the significant increase in malondialdehyde, carbonylated proteins, and oxidized albumin levels found in NDD patients compared to their controls indicates a relationship between lipid peroxidation induced by oxidative stress and the development of neurodegenerative disorders [26]. 4-hydroxy-*trans*-2,3-nonenal has the highest reactivity and hippocampal cytotoxicity and can accumulate in significant amounts in the brain and cerebrospinal fluid of Alzheimer’s and Parkinson’s patients [27, 28].

Oxidative stress is considered an important cause of both forms of Parkinson’s: the inherited and sporadic forms [17]. A high level of oxidized lipids, proteins, and DNA was found in the biological material of Parkinson’s patients, as well as decreased levels of reduced glutathione [29-31], which leads to the generation of more reaction-capable species mediated by the Fenton’s and Haber-Weiss reactions. Overproduction of reactive oxygen forms leads to the degeneration of dopaminergic neurons and, as a consequence, to the development of key symptoms of Parkinson’s, including muscular rigidity, bradykinesia, resting tremor, and postural instability. Thus, patients with Parkinson’s show a 80–90% loss of dopaminergic neurons in substantia nigra and a 40–50% loss of the ventral tegmental area [32].

The possibility of using antioxidants in the treatment of neurodegenerative diseases was confirmed in the end of the last century, but new neuroprotectors are now actively sought among the compounds that inhibit oxidative processes. Vitamin E utilization in the therapy of Alzheimer’s patients at 2000 IU a day for 2 years attenuates the functional decrease of cognitive functions [33]; similarly, administration of this antioxidant at an early age can potentially reduce the risk of Parkinson’s [34]. Another known free radical acceptor is Vitamin C, which protects membrane phospholipids from peroxidation and participates in catecholamine biosynthesis [35]. Despite the fact that ascorbic acid is not a direct scavenger of lypophilic radicals, it has a synergic effect when combined with vitamin E [36, 37]. Resveratrol is a naturally occurring phytoalexin that has the ability to capture active oxygen species, acting as a metal chelator and enzymatic activity modulator [38, 39]. Its antioxidant properties include effective inhibition of lipid peroxidation in the hippocampus and are confirmed by an increased catalase activity [38]. It has also been shown that the extract derived from the leaves of the Chinese Ginkgo tree (*Ginkgo biloba *L.), which has some of the most potent antioxidant properties, can improve cognitive brain functions in the Alzheimer’s disease by reducing the toxicity of Aβ-plaques [40].

The positive impact of the antioxidant compounds used as neuroprotectors was also confirmed by studies of a natural compound derivative representing the alkaloid-derived adducts securinine and tryptamine and also known as allomargaritarine. A study of the neuroprotective properties of this conjugate in various neurotoxicity models using a primary culture of the rat cortex showed that allomargaritarine has a pronounced cytoprotective effect that contributes to an increased cell survival rate after glutamate, Fe3+ and Aβ exposure. The ability of allomargaritarine to protect neurons from death correlated with its antioxidant potential: namely, there was a concentration-depended inhibition of lipid peroxidation caused by ferric iron ions and tert-butylhydroxyperoxide [41, 42]. Allomargaritarine also has an anticonvulsant activity [43], which may be due to its antioxidant potential, since oxidative stress is known to be involved in the pathogenesis of epilepsy [44, 45]. Antioxidant properties are considered one of the mechanisms of the neuroprotective action of one of the bioisosteric analogues of cinnamon acid. Moderate inhibition of rat brain homogenate peroxidation was shown, and, importantly, there was an increased cell survival count of human neuroblastoma SH-SY5Y in ionomycin-induced neurotoxicity [46]. When assessing the effect of structural analogues of Dimebon (derivatives of tetrahydro-gamma-carboline derivatives) on the ratio of reduced and oxidized glutathione, it turned out that DF-407 effectively inhibited the accumulation rate of reactive oxygen species and increased the GSH/GSSG ratio, which indicates a possible effect on the cell defence system and correlates with a decrease of the glutamate-induced death rate of cortical neurons in the brain of new born rats [47]. Therefore, the key role that oxidative stress plays in the development of neurodegenerative diseases, as well as the positive results achieved through the use of antioxidants as potential neuroprotectors, suggests that manipulation of the levels of reactive oxygen species can be considered as a promising means for treating neuropathologies and alleviating their accompanying symptoms.

**Neuroinflammatory reactions in a neurodegenerative process**

Neuroinflammation is a pathological process which is typical of a number of neurodegenerative diseases such as Parkinson’s, Alzheimer’s, amyotrophic lateral sclerosis, and Huntington’s disease. Many of these disorders are proteinopathies and are characterized by an accumulation of specific protein deposits, in particular Aβ in Alzheimer’s [48], resulting in the activation of immunocompetent brain cells and subsequent inflammatory reactions [49, 50]. Thus, it has been shown that activated cells can both reduce the amount of Aβ and increase its toxic effect [48, 51, 52].

The main residents of the immune system in the brain are microglial cells and astrocytes, which participate in the immediate inflammation response. Neuroprotective microglial functions are present in transgenic mice expressing human *APP *under the control of the Thy-1 promoter (APP23) [53]. Moreover, CX3CR1-CX3CL1 receptors play an important role in the interaction between glial cells and neurons. The chemokine receptor CX3CR1 allows microglia to participate in synapse formation and decreases the Aβ level [54, 55]. The expression of the Toll-like receptors TLR-2 and TLR-4 by microglial cells also promotes the uptake of aggregated Aβ [56]. While investigating the role of the chemokine receptor CX3CR1 recruiting glial cells in the pathogenesis of neuroinflammation in animal models, S. Hickman *et al. *noted that the concentration of aggregated Aβ and a number of senile plagues in brain tissues were lower in heterozygous APP/PS1 mice (PS1-APP-CX3CR1+/−). Moreover, unlike APP/PS1 mice, the levels of Aβ lysing enzymes were significantly higher in the animals [57].

Considering the neuroprotective role of astrocytes, it should be mentioned that the proinflammatory cytokines TNF-α, TGF-β, and IL-1β are released by cells at an early response, they subsequently activate adjacent microglial cells, and also degrade soluble Aβ with the help of apolipoproteins and, to a larger extent, ApoE2. Therefore, it is believed that astrocytes can act as a therapeutic target in Alzheimer’s [51]. Yet, neuroinflammation primarily disrupts the cytokine balance and changes the microenvironment; hence, some glial cells may have a pro-inflammatory function. This is due to the synthesis of proinflammatory cytokines (IL-1β, IL-6, TNF-α), the toxic effect of Aβ itself, and the suppression of the phagocytic microglia function in the brain of patients with Alzheimer’s [58, 59, 60]. It was also shown that the glia surrounded by the aggregated amyloid migrates to the amyloid-free regions, skipping its activation and, as a result, their ability to degrade amyloid decreases. [60, 61].

The pathological effects of astrocytes in the brain of patients with Alzheimer’s are caused by the impaired calcium exchange [62], the enhanced glutamate secretion [63] which leads to excitotoxicity, as well as the toxicity of apolipoprotein isoforms (ApoE3, ApoE4) [64]. In general, with the development of amyloidosis, activated astrocytes can both stimulate the neuroprotective functions of microglia at the early stages of Alzheimer’s and suppress the activity of glial cells during the disease.

Current findings on the participation of glia cells in neuroinflammation fit into a polar model reflecting the differentiation of the activated macrophages M1 and M2 in the development of tissue inflammation. However, numerous studies show that this analogy does not describe the complex interaction in the microglia and the neuronal environment [65]. Yet, microglial cell phenotypes appear to be more diverse than expected, which is confirmed by ultrastructural analyses [66]. It is also known that glial activity depends on gender, age, and genotype [67]. Currently, five clusters of cells can be distinguished as participating in the pathogenesis of neurodegenerative diseases [68]. A hypothesis has also been formulated on the transcriptional shift mechanism of microglial cells, which highlights the transcription factors mediating neuroinflammation (NF-κB, Activator protein-1, Interferon regulatory factors, p53 tumor suppressor, and STAT), supporting healthy microglia (PU-1, SALL1, MAFB), and the main factors necessary for cell survival and differentiation [69].

The last identified cluster seems more significant, and it is specified as DAM, the disease-associated microglia. The cells in this cluster are located near amyloid deposits and have a characteristic gene expression, and they contribute to pathological processes, especially at early stages of the disease [70]. The TREM2 receptor (Triggering Receptors Expressed on Myeloid cells) plays a critical role in DAM cluster activation [71] and can be used as a biomarker of an early stage of Alzheimer’s [72]. Thus, TREM2 inhibition, a decrease of variability or the receptor’s knockdown in animal models reduce the likelihood of the disease, phagocytic activity of microglia, as well as total activation and secretion of excitotoxic ApoE isoforms [70, 73, 74, 75].

Neuroinflammation is a complex and multifactor process where the activation of glial cells represents only an aspect of the pathological state in proteinopathies. Inflammatory processes in the brain are not only affected by the microenvironment. T-helper cells are also engaged in the process, which is evidenced in App- Tg mice and in patients with Alzheimer’s [76, 77, 78]. The intestinal microbiota is also involved [79, 80, 81]. The function of the blood-brain barrier is impaired during acute and chronic inflammation. Matrix metalloproteinases (MMP-3, MMP-9), which are involved in the development of pro-inflammatory reactions, play a critical role in the molecular mechanisms of neuroinflammation pathogenesis [82, 83, 84].

euroinflammation is associated with neuronal loss in Parkinson’s disease, which is typically under the control of microglia. Microglial activation in the substantia nigra was found in patients both with sporadic [85] and familial Parkinson’s forms [86], as well as in the substantia nigra and striatum of transgenic animals modelling this pathology, as induced by an inhibitor of complex I of the respiratory chain I complex 1-methyl-4-phenyl-1,2,3,6-tetrahydropyridine (MPTP) [87]. The chronically activated or overactivated microglial condition causes redundant and uncontrolled neuroinflammatory reactions due to an abundant release of free radicals, which, in turn, leads to a self-maintained neurodegeneration cycle [88]. The molecules released from the damaged dopaminergic neurons due to impaired metabolic activity dopamine and reactive microgliosis include neuromelanin, α-synuclein, and the active form of metalloproteinase-3 (MMP-3) [17]. Insoluble extraneuronal neuromelanin granules are found in patients with juvenile idiopathic Parkinson’s [89] and in patients with MPTP-induced parkinsonism [90]. Intracerebral neuromelanin injection causes strong microglial activation and loss of dopaminergic neurons in substantia nigra [91]. Since neuromelanin remains in the extracellular space for a very long time [90], it is considered a target molecule responsible for the triggering of a chronic neuroinflammation in Parkinson’s disease [17]. The addition of aggregated human α-synuclein to a primary culture of mesencephalic neurons induced microglial activation and neurodegeneration, and the cytotoxicity was not observed in the absence of microglia [92]. Moreover, α-synuclein obtained from these neurons stimulated astrocytes to produce inflammation modulators which enhanced the activation of microglia, chemotaxis, and the pro liferation of neuronal cells [93]. Gao *et al. *have shown that transgenic mice expressing mutant α-synuclein develop a persistent neuroinflammation and chronic progressive degeneration of the nigrostrial dopamine pathway initiated by low liposaccharide levels [94]. Moreover, in response to the oxidative stress in dopaminergic neurons, the active form of MMP- 3 causes the activation of microglial cells, which, in turn, leads to the formation of reactive oxygen and nitrogen species [95-99]. MMP-3 also affects protease-activated receptors, their cleavage, the removal of the N-terminal domain, and conversion of the remaining C-terminal domain into the binding ligand, which, in turn, generates intracellular signals and activates microglia [100, 101, 102]. MMP-3 also participates in the formation of interleukin-1 beta (IL- 1β) and facilitates the expression of inflammatory cytokines in activated microglia [84, 103, 104]. Thus, it has been shown that modulation of the various pathways linked to neuroinflammation can considerably contribute to the neuroprotective action of multifunctional drugs.

**Role of mitochondrial stress in neurological disorders**

Despite the fact that the aetiology of many neurodegenerative diseases remains largely unclear, over the last three decades the contribution of mitochondria to the development of neuropathologies has been vigorously discussed, and the accumulated evidence suggests that the dysfunction of these organelles plays an important role in the pathogenesis of a number of NDDs. Mitochondria are the most important components of eukaryotic cells, as they provide high-energy phosphates and products of intermediary metabolism, support homeostasis by participating in the regulation of the electrolyte balance, and maintain the concentration of calcium ions. Mitochondria regulate the production of the reactive oxygen species playing a key role in the initiation of apoptotic cell death; hence, their dysfunction can contribute to the development of a number of neurodegenerative diseases, including Alzheimer’s [105, 106, 107].

Evidence to support this hypothesis has been obtained in studies describing mitochondrial dysfunction (change in morphology and suppression of metabolic activity) correlated with a decrease in ATP production and an increase in the level of reactive oxygen species in the brain [107-111], fibroblasts, and the blood cells [112, 113] of patients with a neurological disorder, as well as in transgenic mice modelling Alzheimer’s [106, 111, 114, 115], and in cell lines expressing the mutant precursor protein amyloid [116]. It is known that in neurodegenerative diseases, numerous mitochondrial dysfunctions are present [117]. Mitochondria undergo several cycles of division and fusion (shortening and elongation), or “mitochondrial dynamics” [118, 119]. The emerging defects in the dynamics of these organelles are associated with the changes in the expression of the fission and fusion proteins determining their morphology [120, 121], as well as the integrity and functional state [120, 122]. Therefore, fine regulation of five basic proteins Drp1, Fis1, Opa1, Mfn1 and Mfn2 controlling the dynamics of mitochondria [123] is necessary to maintain normal functioning of these organelles in brain cells. A postmortem analysis of the brain samples of patients with Alzheimer’s revealed an impaired expression of these genes and, consequently, a change in the morphology of mitochondria compared to healthy patients [124]. These results were also confirmed in studies of a M17 neuroblastoma cell line overexpressing the mutant APP, where changes in the mitochondrial structure were also observed [113], while changes in the morphology of cortical mitochondria in elderly monkeys correlated with increases in active oxygen forms and memory impairment [125].

**Fig. 3:**
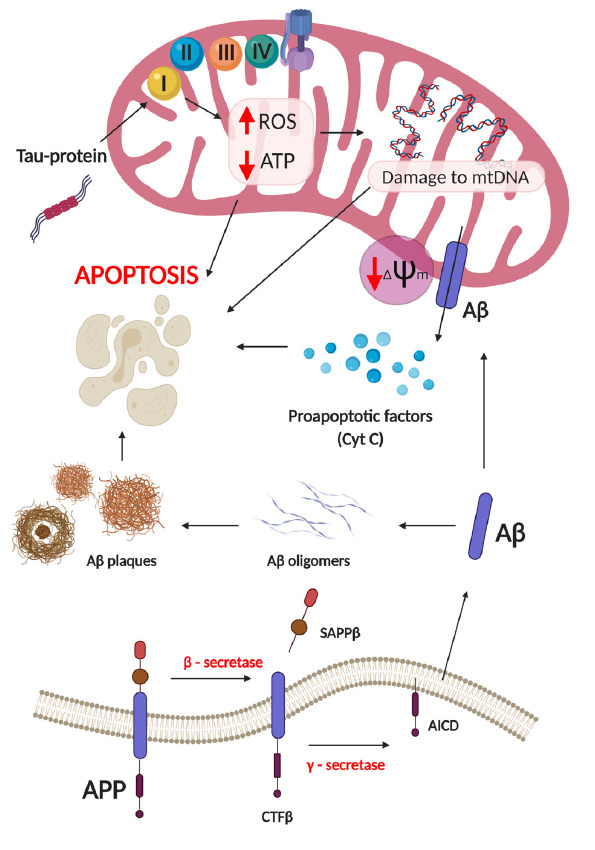
The role of mitochondria and oxidative stress in the development of Alzheimer’s. Mitochondrial dysfunction caused by the action of the pathological tau-protein and β-amyloid isoforms leading to respiratory chain disruption, damage to mtDNA, ROS overproduction, reduction in ATP levels, and a cascade of apoptotic death of nerve cells

Defects in mitochondria bioenergetics manifest themselves in a disruption of the functioning of the electron transport chain, mitochondrial depolarization, increased production of reactive oxygen species, and reduced production of ATP. The respiratory chain localized in the inner mitochondrial membrane is one of the main functional and structural parts of the organelles [126], which catalyses the formation of ATP from ADP and inorganic phosphate via electron transfer between its subunits [127] and, therefore, is considered the most important and indispensable source of energy in mammalian cells. This process also leads to the formation of free radicals [128], resulting in the production of 1–5% of total cell ROS under normal physiological conditions [129]. These by-products of mitochondrial respiration [130] serve as important redox messengers in the regulation of various signalling pathways [17]. However, disruptions in the activity of even one of the electron transport chain complexes of mitochondria (mainly the I and IV complexes) can lead to an overproduction of superoxide radicals and other reactive oxygen species because of intensive reduction in oxygen molecules [131, 132, 133], which, in turn, contributes to the development of oxidative stress, irreversible damage to cell components and, as a consequence death of the cell through mitochondrial apoptosis [134, 135]. As a result, disruption enhances neuronal dysfunction and leads to neurodegenerative disorders [136]. Mitochondrial dysfunctions may be due to the action of a pathological Aβ peptide which destabilizes membranes and penetrates mitochondria through translocases of the outer (TOM) and inner membranes (TIM), resulting in the release of apoptogenic factors, in particular cytochrome c, and subsequent caspase activation and apoptotic cell activation [137]. The dysfunction can also be due to tau [138, 139]. The effects of tau on the mitochondrial functions and dynamics was investigated in neuroblastoma cells expressing a pathological isoform of tau (P301L), which leads to a deficiency in complex I of the respiratory electron transport chain – NADH-ubiquinone oxidoreductase, resulting in a decrease in ATP levels and increased susceptibility to oxidative stress. In addition, increased expression of P301L in neuroblastoma cells also leads to a decreased mobility of mitochondria and their perinuclear clustering, resulting in an activation of the Bax proteins that increase the permeability of the outer membrane of mitochondria and cause apoptosis [140]. However, it is hard to ignore the fact that mitochondrial dysfunction can also precede the formation of pathological Aβ, after which the latter, in an aggregated state, penetrates the membranes of organelles and contributes to a further disruption of their functioning [141]. *Figure 3* outlines the role of mitochondria and oxidative stress in the development of Alzheimer’s.

**Fig. 4:**
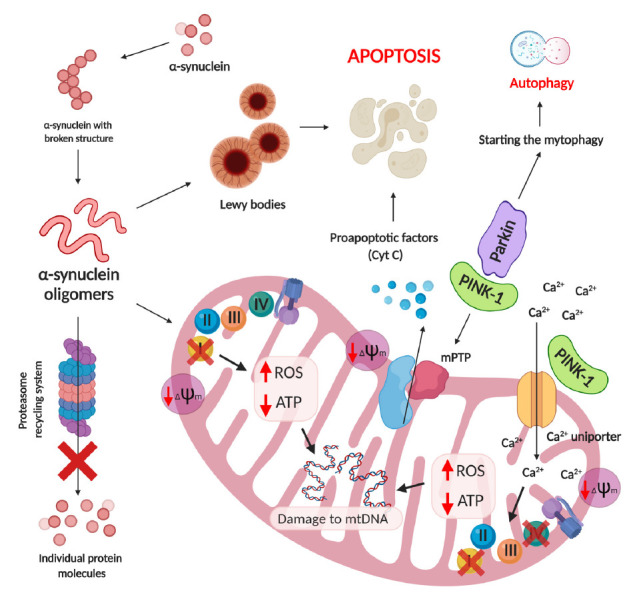
The role of mitochondrial dysfunction in the development of Parkinson’s. Mitochondrial dysfunction caused by overexpression of pathological α-synuclein, mutations in mitochondrial genes and calcium dysregulation lead to changes in the functioning of the electron transport chain complexes, ROS overproduction, a decrease in ATP levels and, as a result, damage to mtDNA and apoptotic neuronal death

Mitochondrial dysfunction also plays a role in the pathogenesis of Parkinson’s (*Fig. 4*). The dopaminergic neurons in the substantia nigra, which are mostly prone to progressive degradation and death in patients with the disease, are very active metabolically and largely depend on energy production as ATP by mitochondria. Any pathological situation leading to mitochondrial dysfunction can induce a significant ROS increase. Overproduction of free radicals initiates the peroxidation of mitochondrial lipids, cardiolipin in particular, and per se leads to cytochrome C release into the cytosol. In turn, this causes apoptosis. As mentioned above, electron leakage after damage to mitochondrial respiratory chain complex I induces ROS generation. Predominant death of dopaminergic neurons was observed following intraperitoneal administration of inhibitors of complex I such as rotenone and 1-methyl-4-phenyl-1,2,3,6-tetrahydropyridine to animals modelling Parkinson’s disease [142]. The level of dopaminergic neurons with impairment of the electron-transport respiratory chain in mitochondria was higher in patients with Parkinson’s than in age-matched controls without any signs of the disease [143]. Enough evidence of the role played by mitochondrial dysfunction and impairment of dopaminergic neurons has been gathered in studies of gene mutations in mitochondrial proteins DJ-1, Parkin, and PINK associated with inherited and sporadic Parkinson’s. The cells obtained from patients with a mutation in the *Parkin *gene show a reduced complex I activation [144]. Parkin-deficient mice present a decreased activity of the respiratory chain in striatum and various types of oxidative damage [145]. *PINK1 *gene mutations induce mitochondrial dysfunction, including formation of abundant free radicals [146]. The sporadic form of Parkinson’s is associated with protein DJ-1, which is a redox-sensitive atypical peroxiredoxin-like peroxidase that eliminates peroxide compounds by self-oxidation. DJ-1 knockout mice accumulate more ROS in brain cells and display a fragmented mitochondrial phenotype [147]. Choi *et al*. have shown that the protein DJ-1 in the brain of patients with Parkinson’s is exposed to oxidative damage [148]. They identified ten different DJ-1 subtypes using 2D gel electrophoresis and mass spectrometry and found that DJ-1 monomers containing acid fragments are selectively aggregated in the frontal cortex of patients. The authors have assumed that oxidative damage to protein DJ-1 can be related to the pathogenesis of the sporadic disease and may be used as a biomarker of an early stage of the disease. An important role in the development of pathology in Parkinson’s disease is assigned to α-synuclein, which is a cytosolic protein that is capable of interacting with mitochondrial membranes and inhibiting complex I of the mitochondrial respiratory chain (*Fig. 4*) [149]. Thus, impairment of the mitochondrial structure and function is found in mice with abundant expression of mutant α-synuclein [150]. It is also likely that calcium dysregulation contributes to oxidative stress and mitochondrial dysfunction in Parkinson’s disease [151, 152]. This is due to the fact that the compact layer of the dopaminergic neurons in the substantia nigra includes L-type ion channels the disruption of which allows extracellular calcium to enter the cytoplasm uncontrollably [153] and thereby enhance dopamine metabolism, shifting the cytosolic concentration of the neurotransmitter to the toxic range of L-DOPA [154]. In particular, Surmeier *et al. *showed that the constant opening of L-type calcium channels in the dopaminergic neurons of the substantia nigra causes oxidative stress, and likewise leads to fluctuations in the mitochondrial potential, which is associated with a disruption of ATP production, which ultimately triggers processes associated with cell death [155]. Isradipine, an L-type calcium channel blocker, can attenuate rotenone-induced dendrite loss (shown in adult midbrain slices), as well as attenuate MPTP-induced neurodegeneration of dopaminergic neurons in mice [156].

Mitochondrial dysfunction leads to a decreased ability by the organelles to regulate intracellular calcium homeostasis and initiate mitochondrial permeability transition [157]. In other words, the increase of the intracellular calcium level can provoke degenerative changes and lead to a significantly higher probability of mitochondrial permeability with subsequent initiation of a cell death cascade via apoptosis and necrosis [158]. Importantly, higher levels of calcium can lead to excess production of active oxygen forms and oxidative stress [159]. The increased calcium levels in the neurons of 3xTg-AD transgenic mice were investigated by Lopez *et al*. [160]. In addition, the mitochondrial dysfunction associated with impaired calcium homeostasis has been described in neurodegenerative pathologies; in particular, in Huntington’s disease [161]. Pronounced defects in calcium regulation were detected in the brain mitochondria of transgenic mice modelling Huntington’s disease, as well as in the lymphblasts of patients with Huntington’s disease [162]. Moreover, the mitochondrial function was also impaired in cell models of the disease [161, 163, 164, 165], whereas the use of mitochondria membrane permeability inhibitors such as Bongkrek acid, Nortriptyline, Desipramine, Trifluoroperazine, and Maprotiline prevented neuronal death and had a neuroprotective effect on animal models of this disorder [163]. Mitochondrial damage is also observed in the neurons of patients with Alzheimer’s, which is accompanied with membrane depolarization, reduced ability to bind Ca^2+^ ions, overproduction of reactive oxygen species and oxidative damage to mitochondrial DNA [166].

The possibility of using mitoprotectors for the treatment of neurodegenerative diseases was also confirmed by the results of studies of bioisosteric analogues of cinnamic acid and polymethoxybenzenes as potential neuroprotectors. A high ability to inhibit calcium-induced opening of the mitochondrial permeability transition pore (over 50%) was established for several compounds. Such mitoprotective activity is considered as a mechanism of the neuroprotective effect of these compounds and correlates with the presence of a cytoprotective potential on a cellular model of neurodegeneration associated with calcium stress in ionomycin-induced neurotoxicity [46]. Such ability was also shown for tetrahydro-gamma-carbolines, structural analogues of Dimebon. These compounds were more likely to inhibit calcium-induced mitochondrial permeability than the drug Dimebon, which reduced the rate of mitochondrial swelling by an average of 20%, whereas the effect of DF-407 was double [167]. Early studies of the effect of tetrahydro-gamma-carbolines on the survival of neurons in the cerebral cortex of newborn rats under glutamate-induced toxicity showed a significant decrease in the death rate of cells treated with these compounds, which may have something to do with their mitoprotective properties [47]. Preincubation of rat mitochondria with allomargaritarine, the conjugate of securinine and tryptamine, inhibits Ca^2+^-induced mitochondrial permeability transition in a dose-dependent manner. It also effectively suppresses it when Aβ35-25 is used as an inducer and, as a result, displays cyto(neuro)protector activity in models of excitotoxicity and toxicity mediated by trivalent iron ions and amyloid [41, 42, 168]. Moreover, allomargaritarine has the ability to reduce Aβ [169]. Therefore, mitochondria represent a promising target in the search for potential neuroprotective agents aimed at preventing or slowing down the development of neurodegenerative diseases: in particular, Alzheimer’s.

**Histone deacetylases (HDACs) as a potential molecular target in the search for neuroprotective agents**

In addition to the main pathological aspects of Alzheimer’s, the formation of toxic β-amyloid aggregates and neurofibrillary tangles, epigenetic regulation mechanisms have now become increasingly important [170, 171]. Epigenetic changes are reversible, do not affect the modifications of primary DNA structure, and can be corrected with pharmacological therapy. Chromosome DNA is enveloped in a compact structure with the specialized proteins called histones. Histones are relatively small proteins with a very large fraction of positively charged amino acids (lysine and arginine); a positive charge helps histones bind to DNA (which is negatively charged) regardless of its nucleotide sequence. Histones perform the two main functions in the cell: they are involved in the packaging of DNA in the nucleus and the epigenetic regulation of transcription, replication, and reparation [172]. Histones undergo post-translation modification by acetylation, deacetylation, phosphorylation, and methylation. Histone acetylation and deacetylation are regulated by histone deacetylases (HDACs) and histone acetyltransferases (HATs) [173, 174]. These processes play a decisive role in the changing of the structure of chromatin and, as a result, regulate gene expression, cell survival, and cell differentiation [175].

**Table:** Classification of histone deacetylases

HDAC family		
Type	Co-factor	Localization
Class I		
HDAC1	Zn^2+^	Nucleus
HDAC2		Nucleus
HDAC3		Nucleus/cytoplasm
HDAC8		Nucleus
Class II		
Subclass IIa		
HDAC4	Zn^2+^	Nucleus/cytoplasm
HDAC5		Nucleus/cytoplasm
HDAC7		Nucleus/cytoplasm
HDAC9		Nucleus/cytoplasm
Subclass IIb		
HDAC6	Zn^2+^	Cytoplasm
HDAC10		Cytoplasm
Class III Sirtuins		
Sir1	Zn^2+^	Nucleus
Sir2		Nucleus
Sir3		Nucleus/cytoplasm
Sir4		Mitochondria
Sir5		Mitochondria
Sir6		Mitochondria
Sir7		Nucleus
Sir8		Nucleolus
Class IV		
HDAC11	Zn^2+^	Nucleus

There are two main subfamilies of HDAC proteins: “zinc-dependent” conventional histone deacetylases and “nicotinamide-adenine-dinucleotide (NAD^+^)-dependent” proteins sirtuins (SIRTs), sometimes referred to as class III HDACs. Depending on their similarity, zinc-dependent HDACs are divided into four different classes (I, II (IIa and IIb), III and IV) which differ in their structure, enzymatic functions, subcellular localization, and expression regions (*Table*) [176]. To date, 18 deacetylases have been identified in mammals. The biological functions of individual HDACs are difficult to establish due to the lack of isoform-specific inhibitors.

**Fig. 5:**
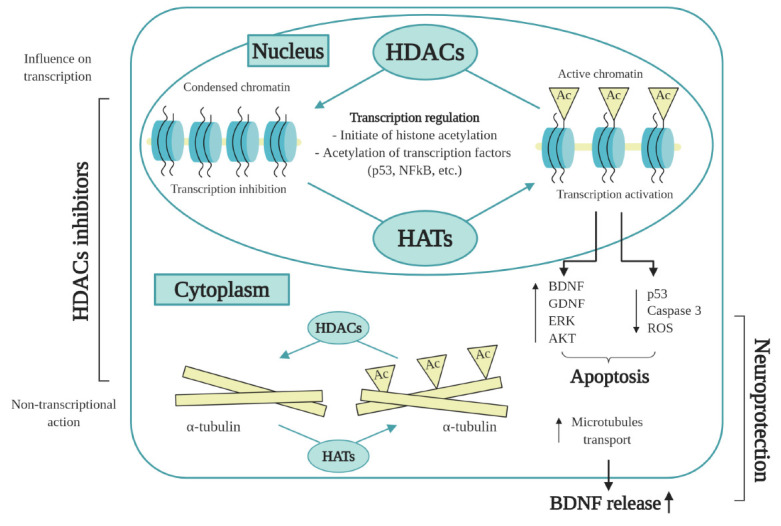
Action of HDAC inhibitors in the cell in neurodegenerative diseases. Impairment of acetylation homeostasis leads to hypoacetylation of histones and, as a result, aberrant transcriptional activity. Inhibition of HDAC activity has transcriptional and non-transcriptional effects. Acetylation of histone proteins in gene promoters, as well as transcription factors, can increase the expression of multiple genes which contribute to neuroprotection, plasticity, and learning/memory. The non-transcriptional action of HDAC inhibitors leads to hyperacetylation and stabilization of microtubule proteins, increase of vesicular transport, and BDNF release

The ratio between the levels of histone acetylase and histone acetyltransferases is strictly regulated in healthy neurons, whereas in neurodegenerative pathologies this ratio is disturbed [177]. HDAC6 is overexpressed in patients with Alzheimer’s, along with the formation of atypical APP, Aβ accumulation, Aβ-mediated hyperphosphorylation of the tau protein, degeneration of cholinergic neurons, and, consequently, severe cognitive decline (*Fig. 5*) [178]. Neurodegenerative diseases are accompanied by dysregulation of transcription, leading to the death of nerve cells; therefore, HDACs are considered very promising targets for the pharmacological correction of neuropathologies [179], in part because of the potential reversibility of such epigenetic modifications [180].

Hahnen *et al*. have considered the involvement of histone deacetylase inhibitors (HDACi) in the regulation of epigenetic events as relates to the development of a number of neurodegenerative processes. Histone deacetylase inhibitors, which were originally used as anti-neoplastic agents, may be effective in neurodegenerative disorders, particularly in Alzheimer’s [181]. The results of numerous studies on the effect of different compounds on HDAC show that the neuroprotective effect of histoneacetylase inhibitors might be attributed to the suppression of Aβ production [182, 183] and, consequently, inhibition of Aβ-induced hyperphosphorylation of the tau-protein [184, 185]. The use of the histone deacetylase inhibitor Entinostat for the treatment of APP/PS1 transgenic mice modelling Alzheimer’s led to an enhanced microglial activation and a decrease in Aβ deposits [186]. The use of suberoylanilide hydroxamic acid (SAHA) in experiments on 20-month-old mice with age-related memory disorders showed spatial memory improvements. At the same time, in elderly mice, a decrease in the level of histone H4K12ac in the hippocampal region of CA1 was established, while SAHA led to the expression of acetylated histones, and also stimulated the activity of NMDA receptors in the hippocampus [187].

Mice overexpressing HDAC2, but not HDAC1, show a decreased synaptic plasticity, in the number of synapses formed, and impaired memory formation, while Vorinostat (an HDAC inhibitor) can restore synaptic plasticity and improve learning and memory [188]. Akhtar *et al*. showed that an increased level of HDAC2 in mature neurons affects the main excitatory neurotransmission, implying the involvement of HDAC2 in synaptic plasticity [189]. McQuown *et al*. found that, in HDAC3-Flox-modified mice (deletion of HDAC3 in the hippocampal region of CA1) or in mice treated with the selective HDAC3 inhibitor RGFP136, the histone acetylation process is enhanced and long-term memory is significantly improved [190]. Moreover, Bardai *et al*. have suggested that HDAC3 is a protein that exhibits its own strong neurotoxic activity, while its toxic effect is cell-selective. HDAC3 is phosphorylated directly by GSK-3β, and inhibition of GSK-3β protects mice from HDAC3-induced neurotoxicity [191]. HDAC6 is localized mainly in the cytoplasm and catalyses a number of non-histone proteins, such as tubulin and deacetylase HSP90 [192, 193].

The level of HDAC6 in the brain of patients with Alzheimer’s is significantly higher in the cortex and hippocampus compared to the brain of healthy people. Tubacin (a selective HDAC6 inhibitor) attenuates the site-specific phosphorylation of the tau-protein [194] and enhances mitochondrial migration in hippocampal neurons. GSK-3β participates in the regulation of HDAC6 activity through its phosphorylation [195]. Selective HDAC6 inhibition ensures protection from the neurodegeneration induced by oxidative stress and contributes to the proliferation of neurites in cortical neurons [196]. HDAC4 can also play a significant role in the functioning of neuronal cells. The enzyme is predominantly found in the cytoplasm of brain cells, and abnormal expression of HDAC4 occurs in the nucleus that contributes to neuronal apoptosis. Its inactivation suppresses cell death [197].

Recent findings have implicated sirtuins in the development of neurodegenerative diseases. A significant decrease in Sir1 was found in the parietal cortex of patients with Alzheimer’s compared to the controls. Therefore, the accumulation of Aβ and tau proteins may be associated with a loss of Sir1 function [198]. In addition, memory and synaptic plasticity impairments are also found in mutant Sir1-deficient mice [199]. Moreover, abundant expression of NAD^+^-dependent deacetylase Sir1 in a mouse model of Alzheimer’s decreases Aβ production and the formation of plagues via the activation of the gene encoding α-secretase *ADAM10 *[200]. Sir3 knockdown increases the generation of mitochondrial reactive oxygen species in fertilized mouse oocytes, and the formation of mitochondrial ROS is accompanied by an increase in the amount of the p53 protein [201]. Moreover, treatment of the primary cultures of neurons in the cerebral cortex of mice with glutamate induces excessive production of ROS, as well as an increase in the level of mitochondrial Sir3, while overexpression of Sir3 significantly reduces the formation of mitochondrial ROS. Apparently, Sir3 is involved in the protection of nerve cells from oxidative stress and excitotoxicity [202].

The accumulated data support the opinion that HDAC proteins are involved in the development of neurodegenerative diseases. HDACs regulate the level of histone acetylation and, as a consequence, affect the expression of some of the genes involved in memory formation, synaptic plasticity, and other processes necessary for the normal functioning of brain cells. HDAC inhibitors can reduce cognitive deficits in animal models with neurodegenerative disorders. HDAC inhibitors can potentially act on suppressing Aβ-induced hyperphosphorylation of the tau protein, as well as in regulating the expression of the genes that are involved in learning and memory (*Fig. 5*). The possibility of pharmacological correction of neurodegenerative diseases using HDAC inhibitors is being considered, but a number of unsolved problems remain. Most current inhibitors of histone deacetylases are pan-selective; i.e., they act against all HDACs types, which causes massive changes in gene expression leading to multiple adverse effects [203], because HDACs participate both in cell survival and death processes. Therefore, in order to develop selective HDAC inhibitors with low toxicity to normal cells, it is necessary to elucidate the exact role of individual members of the HDAC family in various neuropathologies.

**Aggregation of pathogenic protein forms as a key target in the search of potential drugs for the treatment of neurological disorders**

The introduction of the latest cell technologies, bioinformatics, and targeted manipulation of the genome of laboratory animals have led to rapid progress in this field and allowed us to design a new classification of the fundamental processes underlying neurodegeneration. As a result, some concepts have been revised and changes in the classification of neurodegenerative diseases have been introduced. It has been established that a wide range of neurodegenerative diseases with different clinical manifestations have a similar molecular mechanism of pathogenesis. This mechanism is based on a pathological aggregation of proteins that leads to the development of proteinopathy [204, 205]. Many neurodegenerative diseases are characterized by the presence of pathological inclusions of various types in tissues of the nervous system [206]. The cascade nature of the complex mechanism of formation of detectable inclusions is revealed, and the molecular-cellular events occurring at the main stages of this pathological process are identified. [207, 208]. For example, in Parkinson’s disease, the *SNCA *gene encoding α-synuclein, a short cytoplasmic protein (140 amino acids in humans), is predominantly synthesized in the nervous system and localized in presynaptic terminals [209, 210, 211]. The most typical histopathological signs of Parkinson’s are the Lewy bodies found in the dopaminergic neurons of substantia nigra and dystrophic neuritis in the tract leading from substantia nigra to the striatum containing aggregates of various proteins [212, 213]. The key role in the formation of these deposits is played by the fibrillar form of α-synuclein, which has unique physical and chemical properties [214, 215]. It should be noted that the formation of Lewy bodies in the neurons of the cerebral cortex also results in diseases that are classified as a separate group of dementia. For example, cytoplasmic and nuclear deposits in neurons and oligodendrocytes form in multiple systemic atrophy [216, 217, 218].

In Alzheimer’s, it has been established that muta tions in three various genes, *APP*, presenilin-1, and presenilin-2 (*PSEN1*, *PSEN2*), lead to the development of hereditary forms of Alzheimer’s with early manifestation (clinical symptoms appear before the age of 65 years) [219, 220]. At the same time, familial and sporadic forms of Alzheimer’s are similar: the nervous tissues of patients contain protein aggregates of two types: amyloid plaques and neurofibrillary tangles, the main components of which are Aβ and hyperphosphorylated forms of the tau protein, respectively. A hypothesis about the transformation of non-toxic Aβ monomers into its toxic oligomers [221], which can interact with several post-synaptic components, including glutamatergic receptors (N-methyl-D-aspartate (NMDA) and metabotropic glutamate receptor 5 (mGluR5)), the prion protein, neurotrophin receptor, and the A7-nicotin acetylcholine receptor [222], and contribute to synaptic damage, is one of the predominant ones seeking to explain the order of pathogenic events leading to neurodegeneration. It is known that oligomers Aβ can form channels. leading to the impairment of membrane permeability and, as a result, calcium homeostasis, which in turn induces neuronal death [223, 224]. Similarly, toxic oligomers Aβ can modulate the activity of NMDA-subtype glutamate receptors [225], attenuate the mGluR-dependent mechanisms [226] inducing the impairment of recirculation of the synaptic glutamate contributing to synapse depression, and damaging synaptic plasticity [227].

It has also been shown that the oligomeric form of Aβ activates extrasynaptic NMDA-receptors in neurons which, in turn, leads to hyperphosphorylation of the tau-protein, activation of caspase-3, production of nitric oxide. and synaptic depression [228], and inhibition of this subtype of glutamate receptors protects synapses from Aβ-induced damage and, apparently, eliminates memory difficulties [229, 230], which clearly confirms the potential existent in using modulators of this process.

Although the exact molecular mechanisms of neurodegeneration development are still unclear, hyper phosphorylation of the tau-protein is one of the key roles in the pathogenesis of this pathology. To a large extent, the tau-protein is involved in the abovementioned processes, acting in parallel or in combination with Aβ [231]. In a model of tau-induced neurodegeneration, it was shown that an abnormally phosphorylated protein initiates the binding and stabilization of filamentous actin, which leads to mitochondrial dysfunction and oxidative stress, DNA damage and, ultimately, apoptosis [232]. Decreased tau protein levels protected both transgenic and nontransgenic mice from excitotoxicity and restored the memory function in a tauopathy model [233].

The tau-protein does not have a rigid three-dimensional structure [234]. However, its shortening and hyperphosphorylation can cause multiple pathological changes in the structure and lead to the formation of insoluble paired helical filaments and larger aggregates [234-238]. First of all, such transformations lead to a loss of the physiological function of the native protein (participation in the assembly of tubulin monomers into microtubules), and secondly, to a toxic effect on brain cells [235, 234].

Because tau plays an important role in the physiological dynamics of microtubules and thus ensures the normal functioning of cells [239], researchers are interested in the development of drugs that can act on this protein. An in-depth study of the molecular mechanisms underlying the pathological transformations of the tau protein opens up the possibility of specifically targeting the pathological modifications of tau for therapeutic purposes. At the moment, there are several approaches for the development of such agents targeting directly or indirectly the tau-protein: compounds which prevent or reverse tau aggregation [240, 241, 242], low-molecular drugs which inhibit kinases or activate tau phosphatases [243, 244], compounds that are stabilizing microtubules [245], drugs which contribute to a proteolytic degradation of incorrectly folded tau-proteins [239, 246, 247] and immunosuppressive agents [234], as well as strategies aimed at active and passive immunization [234, 248, 249].

It has been shown that monoclonal antibodies can differentiate between tau-protein isoforms and have a different effect on native than transformed proteins. Taniguchi *et al. *demonstrated that the monoclonal antibodies RTA-1 and RTA-2 binding specifically to the R1 and R2 parts of tau prevent the formation of spiral filaments *in vitro *and simultaneously stimulate tubulin assembly induced by tau [250]. At least three vaccines acting on different pathogenic forms of Aβ are in clinical studies. At the same time, there are currently no data on the results of these trials. In the transgenic APP animals modeling Alzheimer’s, the effectiveness of active immunization was clearly shown, which leads to a decrease in Aβ deposits and, as a result, alleviates the associated brain damage [251, 252, 253]. Asuni *et al*. demonstrated that hat active immunization with the epitope of a phosphorylated tau protein of transgenic mice expressing the P301L mutant tau in neurons reduces the amount of aggregated protein in the brain and slows down the progression of the behavioural phenotype associated with this pathology [254, 255]. Furthermore, a significant correlation was observed between motor activity values obtained in the behavioural analysis and the tau pathology in the excitable area of the cortex and brain stem, which play an evident role in motor coordination. It shows a direct correlation between the main pathological feature of the model and the related functional disorders [255] and states that immunotherapy approaches targeting the pathological tau-protein form represent a promising approach to the treatment and/or diagnosing of various tauopathies, and the Alzheimer’s disease in particular.

Some other diseases can be compared in a similar way. In amyotrophic lateral sclerosis, the autopsy material of patients showed deposits containing the proteins FUS, TDP-43, OPTN, UBQLN2, as well as products of intron repetition translation in the *C9ORF72 *gene [256, 257], while polyglutamine deposits had accumulated in neurons in patients with Huntington’s disease as a result of the expansion of trinucleotide CAG-repetition in the huntingtin gene [258, 259]. Despite the difference in the functions of pathogenic proteins, susceptibility to aggregation is the fundamental feature of a wide range of neurodegenerative diseases; therefore, the aggregation of pathogenic protein forms can be considered as the key therapeutic target.

## CONCLUSION

Our investigations of the multiple hypotheses put forth in the attempts to accurately identify the specific source of any neurodegenerative disorder failed to pinpoint any primary cause. Therefore, it appears necessary to take into account multiplicity (combination) in the context of aetiology of neurodegenerative diseases. This should be the case when a set of mutations or factors, ranging from neuroinflammatory processes to the aggregation of proteins in neuronal cells, leads to the sequential accumulation of a whole tangle of molecular pathologies. The foundational aspect in the development of new drugs should rests in a multifactorial nature of their therapeutic effect. Such drugs should have a multitarget purpose, even if they have no or little significant impact on any of the listed molecular targets. They should affect as many targets as possible. Given the hardly conclusive, and sometimes controversial, studies that aim to identify the root causes of neurodegenerative diseases, it appears that we are only now starting to understand the key factors whose combination triggers a neurodegenerative process.

## Abbreviations

DAM: disease-associated microglia
HATs: histone acetyltransferases
HDACi: histone deacetylase inhibitors
HDACs: histone deacetylases
mGluR5: metabotropic glutamate receptor 5
MMP-3, MMP-9: matrix metalloproteinase 3, 9
NDD: neurodegenerative diseases
NMDA: N-methyl-D-aspartate
PSEN1, PSEN2: presenilin 1, 2
ROS: reactive oxygen species
SIRTs: sirtuins
TIM: translocase of the inner membrane
TOM: translocase of the outer membrane
TREM2: triggering receptors expressed on myeloid cells 2
